# Lymph node ratio-based staging system as an alternative to the current TNM staging system to assess outcome in adenocarcinoma of the esophagogastric junction after surgical resection

**DOI:** 10.18632/oncotarget.11188

**Published:** 2016-08-10

**Authors:** Hongdian Zhang, Xiaobin Shang, Chuangui Chen, Yongyin Gao, Xiangming Xiao, Peng Tang, Xiaofeng Duan, Mingjian Yang, Hongjing Jiang, Zhentao Yu

**Affiliations:** ^1^ Department of Esophageal Cancer, Tianjin Medical University, Cancer Institute and Hospital, Key Laboratory of Cancer Prevention and Therapy of Tianjin City, Tianjin, China; ^2^ Department of Cardiopulmonary Function, Tianjin Medical University, Cancer Institute and Hospital, Key Laboratory of Cancer Prevention and Therapy of Tianjin City, Tianjin, China; ^3^ Department of General Surgery, Weifang People's Hospital, Shandong, China

**Keywords:** adenocarcinoma of the esophagogastric junction, lymph node metastasis, metastatic lymph node ratio, tumor-N-ratio-metastasis (TrNM) staging system, prognosis

## Abstract

This study aimed to assess the prognostic value of the hypothetical tumor-N-ratio (rN)-metastasis (TrNM) staging system in adenocarcinoma of the esophagogastric junction (AEG). The clinical data of 387 AEG patients who received surgical resection were retrospectively reviewed. The optimal cut-off point of rN was calculated by the best cut-off approach using log-rank test. Kaplan-Meier plots and Cox regressions model were applied for univariate and multivariate survival analyses. A TrNM staging system based on rN was proposed. The discriminating ability of each staging was evaluated by using an adjusted hazard ratio (HR) and a −2log likelihood. The prediction accuracy of the model was assessed by using the area under the curve (AUC) and the Harrell's C-index. The number of examined lymph nodes (LNs) was correlated with metastatic LNs (r = 0.322, *P* < 0.001) but not with rN (*r* = 0.098, *P* > 0.05). The optimal cut-points of rN were calculated as 0, 0~0.3, 0.3~0.6, and 0.6~1.0. Univariate analysis revealed that pN and rN classifications significantly influenced patients’ RFS and OS (*P* < 0.001). Multivariate analysis adjusted for significant factors revealed that rN was recognized as an independent risk factor. A larger HR, a smaller −2log likelihood and a larger prediction accuracy were obtained for rN and the modified TrNM staging system. Taken together, our study demonstrates that the proposed N-ratio-based TrNM staging system is more reliable than the TNM staging system in evaluating prognosis of AEG patients after curative resection.

## INTRODUCTION

The adenocarcinoma of the esophagogastric junction (AEG) is a special clinical disease with different risk factors, unique clinicopathological characteristics and biological behaviors; this condition is characterized by pathologically different tumors developing in the border between the esophageal squamous epithelium and the gastric adenomatous epithelium [1]. Despite the improvements in multimodal treatment strategies in the past decades, the prognosis of AEG remains poor [2].

Lymph node metastasis has been considered as the foremost factor in determining the prognosis of AEG patients [3]. However, the number of involved LNs relies heavily on the number of removed LNs from each patient. If the number of retrieved and examined LNs is small, down migration of the pN stage may occur. Conversely, if the number is large, up migration of the pN stage may be observed [4, 5].

In order to overcome these limitations, a new modified prognostic tool incorporating the ratio between metastatic and dissected LNs has been proposed by several authors, which can more accurately reflect the degree of lymph node metastasis and reduce stage migration [4, 6]. Although the prognostic significance of rN in esophageal and gastric cancers has been extensively investigated, this factor in AEG patients has been rarely examined; and whether the rN classification is more optimal for prognosis than the pN classification remains unknown [7, 8].

Recently, some studies have indicated that the tumor-ratio-metastasis (TrNM) staging system based on N-ratio can be used as an alternative to the traditional TNM staging system for the prognostic evaluation of tumors. However, to the best of our knowledge, the prognostic significance of the hypothetical TrNM staging system has not yet to be formally investigated in AEG. Hence, in the present study, we investigated whether patients with AEG can be classified into meaningful risk categories on the basis of rN. And the prognostic power of the TrNM staging system was evaluated by comparing it with the current TNM staging system for AEG patients after surgical resection.

## RESULTS

### Clinicopathological parameters of patients

The baseline characteristics of the 389 AEG patients who met the inclusion criteria are shown in Table 1. There were 292(75.1%) males and 97(24.9%) females. The median age was 62 years, ranging from 22~86 years. The median value of tumor size was 5.0 cm (range, 0.5~12.0cm). With respect to surgery, 306 patients underwent curative proximal gastrectomy, 83 cases underwent total gastrectomy. A total of 6791 lymph nodes were picked up and histologically examined, with an average of 17.5 (median 15, range, 4~71) per case. According to histopathological grading, differentiated tumors were observed in 211 (54.2 %) patients, and undifferentiated tumors in remaining 178 (45.8 %) patients.

**Table 1:** Univariate survival analysis of clinicopathologic factors associated with RFS and OS in 389 AEG patients after curative surgery

Clinicopathologic factors	Cases	Recurrence-free survival			Overall survival		
		5-YSR (%)	*x* ^2^	*P* value	5-YSR (%)	*x* ^2^	*P* value
Gender			1.553	0.213		2.536	
Male	292	24.7			28.2		0.111
Female	97	28.9			36.6		
Age (years)			0.618	0.414		0.678	
<65	241	26.3			31.8		0.410
≥65	148	24.2			27.8		
Tumor size (cm)			4.974	**0.026**		4.790	**0.029**
≤5	222	28.4			33.7		
>5	167	22.2			25.6		
Borrmann classification			4.142	**0.042**		6.200	**0.013**
I, II	161	31.1			36.6		
III, IV	228	21.9			25.8		
Laurren type			3.015	0.081		4.775	**0.029**
Intestinal type	154	30.1			37.3		
Non-intestinal type	235	20.6			26.0		
Histopathological grading			7.741	**0.005**		5.065	
Differentiated	211	31.8			35.6		**0.024**
Undifferentiated	178	18.5			23.6		
AJCC pT classification			32.686	**0.000**		28.897	**0.000**
pT1	10	80.0			90.0		
pT2	48	43.7			49.9		
pT3	26	40.3			43.3		
pT4	305	19.7			24.0		
AJCC pN classification			**68.979**	**0.000**		**63.895**	**0.000**
pN0	135	40.7			47.7		
pN1	97	26.8			31.4		
pN2	84	17.9			19.7		
pN3	73	5.5			7.0		
rN classification			**110.179**	**0.000**		**107.002**	**0.000**
rN0	135	40.7			47.7		
rN1	127	25.9			30.2		
rN2	73	13.7			17.3		
rN3	54	1.9			2.5		

AEG, adenocarcinoma of the esophagogastric junction; RFS, recurrence-free survival; OS, overall survival;

5-YSR, 5-year survival rate; pN, lymph node status; rN, N-ratio.

Based on the criteria of the 7th edition of the UICC/AJCC TNM staging system, 135(34.7%) patients were classified as pN0, 97 (24.9%) as pN1, 84 (21.6%) as pN2 and 73 (18.8%) as pN3, respectively. With regarding to TNM staging, 36 patients were at stage I, 125 patients were at stage II, 82 patients were at stage IIIA, 78 patients were at stage IIIB, and 68 were at stage IIIC.

### Correlation analysis among metastatic lymph nodes, rN, and removed lymph nodes

**Figure 1:**
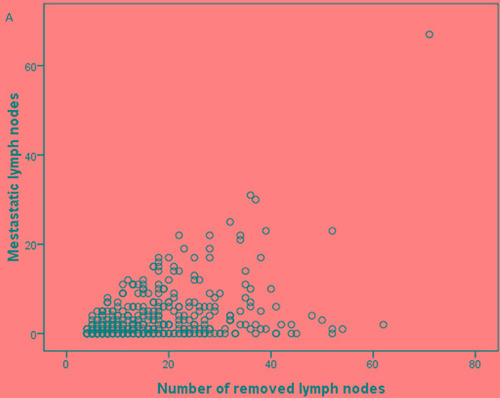

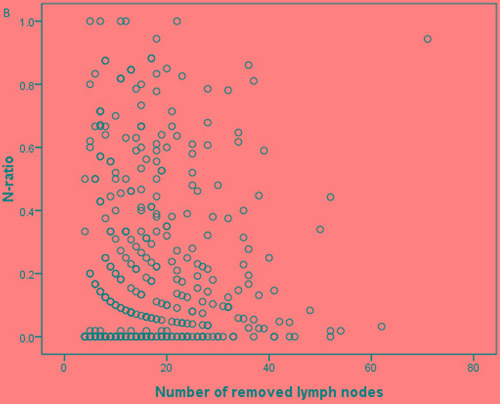

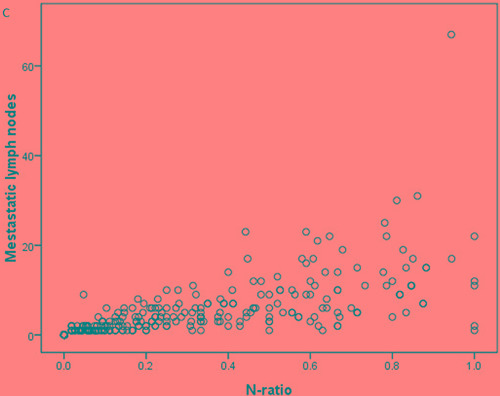
The correlation analysis between (A) the number of removed lymph nodes and metastatic lymph nodes; (B) the number of removed lymph nodes and N-ratio; (C) the number of metastatic lymph nodes and N-ratio

As expected, there was a positive correlation between the number of metastatic LNs and removed LNs according to the Spearman's correlation test (*r* = 0.322, *P* < 0.001, Figure 1A). However, the rN was not associated with the number of removed LNs (*r* = 0.098, *P* > 0.05, 1b). Furthermore, the correlation between rN and the number of metastatic LNs was significant (*r* = 0.929, *P* < 0.001, 1c). These results demonstrated that rN was not influenced by surgery, however, pN was influenced by surgery.

### Re-classification of N-ratio Categories

To determine the appropriate cut-points of the rN value that determine the greatest actuarial survival difference among the resulting subgroups in the entire cohort, our analysis was conducted as follows: firstly, patients having no involved LNs (rN = 0) were assigned to one group because it has been well known that their prognosis was significantly better than patients with metastatic LNs [9]. Furthermore, we determined another two appropriate cut-off points for categorizing the rN to make it comparable with the AJCC pN classification. The patients were stratified into 10 subgroups based on 0.1 intervals of rN. As there were few cases in the 0.7, 0.8, 0.9 or 1.0 rN subgroups, the 0.7 and 0.8 subgroups, and the 0.9 and 1.0 subgroups were combined. According to the log-rank test, the another two optimal cut-off points 0.3 and 0.6 were chosen for subsequent analysis. Finally, the rN intervals were categorized as rN 0 (0%), rN 1 (1%~30%), rN 2 (31%~60%) and rN 3 ( > 60%).

Accordingly, a novel TrNM staging was established: 38 patients were at stage I, 125 patients were at stage II, 115 patients were at stage IIIA, 58 patients were at stage IIIB, and 53 patients were at stage IIIC.

### Univariate and multivariate survival analysis

The 5-year RFS and OS rates for the whole group of patients were 25.7%, 30.3%, the median RFS and OS time were 31 and 38 months, respectively.

**Figure 2:**
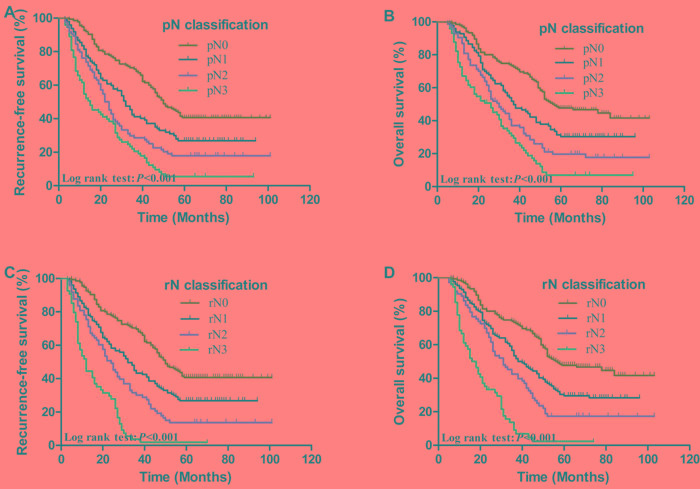
Kaplan-Meier survival curves of recurrence-free survival and overall survival in patients with AEG according to pN and rN classifications **A.**, **B.**, effect of pN classification; **C.**, **D.**, effect of rN classification (P-values were calculated by the log-rank test)

The 5-year RFS and OS curves of patients based on the pN and rN classification are shown in Figure 2. The 5-year RFS rates of patients with pN0, pN1, pN2 and pN3 were 40.7%, 26.8%, 17.9% and 5.5%, respectively (*P* < 0.001, 2A). The 5-year OS rates of patients with pN0, pN1, pN2 and pN3 were 47.7%, 31.4%, 19.7% and 7.0%, respectively (*P* < 0.001, 2B). The 5-year RFS rates of patients with rN0, rN1, rN2 and rN3 were 40.7%, 25.9%, 13.7% and 1.9%, respectively (*P* < 0.001, 2C). The 5-year OS rates of patients with rN0, rN1, rN2 and rN3 were 47.7%, 30.2%, 17.3% and 2.5%, respectively (*P* < 0.001, 2D).

Univariate and multivariate analyses were applied to evaluate clinicopathological variables relating to 5-year RFS and OS. The results of univariate analysis are shown in Table 1. For RFS, factors significantly influencing 5-year RFS were tumor size (*P* = 0.026), Borrmann type (*P* = 0.042), histopathological grading (*P* = 0.005), pT classification (*P* < 0.001), pN classification (*P* < 0.001), and rN classification (*P* < 0.001). For OS, variables significantly associated with 5-year OS were tumor size (*P* = 0.029), Borrmann type (*P* = 0.013), Lauren classification(*P* = 0.029), histopathological grading (*P* = 0.024), pT classification (*P* < 0.001), pN classification (*P* < 0.001), and rN classification (*P* < 0.001).

Multiple survival analysis was performed by the Cox's proportional hazard model to identify the independent prognostic factors. We firstly set up a model including all the significant parameters in the univariate analysis as well as pN classification, except for rN classification. As a result, pT classification (RFS: *P* < 0.001; OS: *P* = 0.001) and pN classification (RFS: *P* < 0.001; OS:*P* < 0.001) were determined to be independent predictors. Similarly, when pN was substituted by rN classification and used for lymph node involvement, this factor (RFS: *P* < 0.001; OS: *P* < 0.001) was identified as an independent prognostic factor in addition to pT classification (RFS: *P* < 0.001; OS: *P* < 0.001). However, when both the pN and rN classification were combined in the model at the same time, rN classification was highly significant (RFS:*P* < 0.001; OS: *P* < 0.001), while pN classification was not a significant factor. These results are shown in Table 2.

**Table 2:** Multivariate analysis of RFS and OS in 389 AEG patients subjected to curative surgery

Clinicopathologic factors	Multivariate Analysis 1		Multivariate Analysis 2		Multivariate Analysis 3	
	HR (95%CI)	*P* value	HR (95%CI)	*P* value	HR (95%CI)	*P* value
**Recurrence-free survival**						
Tumor size	1.041 (0.819~1.325)	0.741	1.043 (0.820~1.326)	0.732	1.034 (0.812~1.318)	0.786
Borrmann classification	1.160 (0.912~1.474)	0.226	1.175 (0.924~1.494)	0.188	1.175 (0.924~1.495)	0.187
Histopathological grading	1.157 (0.914~1.464)	0.214	1.150 (0.928~1.487)	0.176	1.151 (0.926~1.485)	0.175
pT classification	1.440 (1.204~1.726)	0.000	1.401 (1.168~1.680)	0.000	1.401 (1.168~1.680)	0.000
pN classification	1.442 (1.293~1.608)	0.000	—	—	1.047 (0.869~1.262)	0.629
rN classification	—	—	1.637 (1.449~1.850)	0.000	1.573 (1.283~1.929)	0.000
**Overall survival**						
Tumor size	1.044 (0.815~1.336)	0.733	1.039 (0.812~1.331)	0.759	1.031 (0.804~1.322)	0.808
Borrmann classification	1.244 (0.970~1.596)	0.086	1.259 (0.981~1.615)	0.070	1.259 (0.981~1.615)	0.070
Lauren type	1.045 (0.803~1.360)	0.741	1.019 (0.783~1.325)	0.891	1.016 (0.781~1.322)	0.905
Histopathological grading	1.063 (0.825~1.370)	0.635	1.097 (0.852~1.413)	0.471	1.094 (0.850~1.409)	0.486
pT classification	1.436 (1.189~1.735)	0.001	1.395 (1.153~1.688)	0.001	1.395 (1.153~1.687)	0.001
pN classification	1.448 (1.288~1.615)	0.000	—	—	1.050 (0.869~1.268)	0.613
rN classification	—	—	1.646 (1.449~1.870)	0.000	1.579 (1.284~1.941)	0.000

AEG, adenocarcinoma of the esophagogastric junction; RFS, recurrence-free survival; OS, overall survival;

HR, hazard ratio; CI, confidence interval; pN, lymph node status; rN, N-ratio.

We used the adjusted hazard ratio (HR) and −2log likelihood to evaluate the discriminating abilities of each staging system. As a result, we found that the HRs for rN (RFS:1.637; OS:1.646) were larger than pN classification (RFS:1.442; OS:1.448). While the −2log likelihood for rN (RFS:3066.658; OS:2879.804) were smaller than pN classification (RFS:3087.341; OS:2899.995). Therefore, we inferred that rN classification may provide a better prognosis estimation than pN classification for AEG patients.

### Survival analysis based on pN classification according to the rN classification

The 5-year RFS and OS rates of patients were compared with different pN when stratifying by rN classifications and with different rN when stratifying by pN classifications. As shown in Table 3, for RFS, there were significant differences in 5-year RFS rates among different rN classifications within the same pN (*P <* 0.05), but not among different pN classifications within the same rN (*P* > 0.05). For OS, there were significant differences in 5-year OS rates among different rN classifications within the same pN (*P <* 0.05), but not among different pN classifications within the same rN (*P* > 0.05).

**Table 3:** RFS and OS rates with different pN classifications stratified by the rN classification

Lymph node classification	rN0		rN1		rN2		rN3		χ2	*P*
	No.	5-YSR (%)	No.	5-YSR (%)	No.	5-YSR (%)	No.	5-YSR (%)		
**RFS**										
pN0	135	40.7	-	-	-	-	-	-	-	-
pN1	-	-	80	28.8%	11	27.3%	6	0	13.202	0.001
pN2	-	-	40	27.5%	33	12.1%	11	0	7.500	0.024
pN3	-	-	7	0	29	10.3%	37	2.7%	9.433	0.009
*χ2*	-		1.520		1.264		0.287			
*P*	-		0.468		0.532		0.762			
**OS**										
pN0	135	47.0	-	-	-	-	-	-	-	-
pN1	-	-	80	33.5%	11	34.1%	6	0	9.617	0.008
pN2	-	-	40	29.6%	33	15.2%	11	0	9.139	0.010
pN3	-	-	7	0	29	13.5%	37	3.9%	13.335	0.001
*χ2*	-		2.508		1.236		0.254			
*P*	-		0.285		0.539		0.881			

RFS, recurrence-free survival; OS, overall survival; 5-YSR, 5-year survival rate;

pN, lymph node status; rN, N-ratio.

### Analyses of the most appropriate prognostic classification for the survival prediction of AEG patients

**Figure 3:**
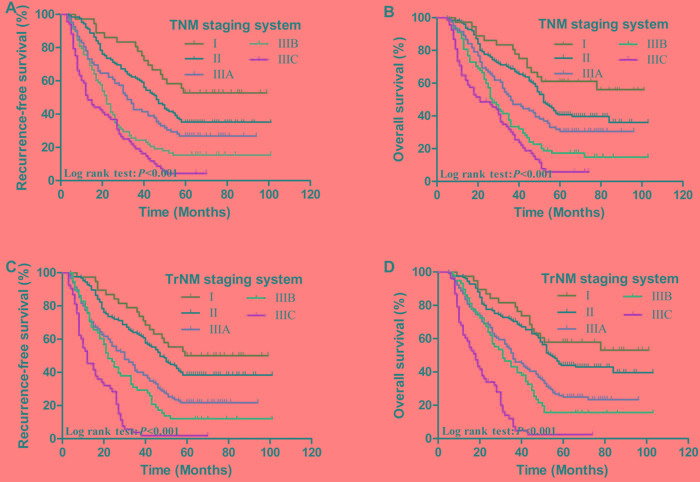
Kaplan-Meier survival curves of recurrence-free survival and overall survival in patients with AEG according to TNM and TrNM staging systems **A.**, **B.**, effect of TNM staging system; **C.**, **D.**, effect of TrNM staging system (P-values were calculated by the log-rank test)

In the TNM staging system, the 5-year RFS rates of patients at stages I, II, IIIA, IIIB and IIIC were 52.8%, 35.2%, 26.8%, 15.4% and 4.4%, respectively (*P* < 0.001, Figure 3A). The 5-years OS rates of patients at stages I, II, IIIA, IIIB and IIIC were 61.1%, 40.7%, 31.8%, 17.3% and 5.8%, respectively (*P* < 0.001, Figure 3B). In the modified TrNM staging system, the 5-year RFS rates of patients at stages I, II, IIIA, IIIB and IIIC were 50.0%, 38.4%, 21.7%, 12.1% and 1.9%, respectively (*P* < 0.001, Figure 3C). The 5-years OS rates of patients at stages I, II, IIIA, IIIB and IIIC were 57.9%, 44.0%, 25.8%, 15.7% and 2.4%, respectively (*P* < 0.001, Figure 3D). These results are listed in Table 4.

**Table 4:** RFS and OS rates according to the TNM and TrNM staging system in 389 AEG patients after curative surgery

Clinicopathologic factors	Cases	Recurrence-free survival				Overall survival			
		5-YSR (%)	χ 2	HR (95%CI)	*P* value	5-YSR (%)	χ 2	HR (95%CI)	*P* value
TNM staging system			**78.935**	**1.495 (1.361~1.643)**	**0.000**		**71.957**	**1.494 (1.355~1.647)**	**0.000**
I	36	52.8				61.1			
II	125	35.2				40.7			
IIIA	82	26.8				31.8			
IIIB	78	15.4				17.3			
IIIC	68	4.4				5.8			
TrNM staging system			**112.373**	**1.647 (1.484~1.829)**	**0.000**		**111.396**	**1.664 (1.492~1.855)**	**0.000**
I	38	50.0				57.9			
II	125	38.4				44.0			
IIIA	115	21.7				25.8			
IIIB	58	12.1				15.7			
IIIC	53	1.9				2.4			

AEG, adenocarc inoma of the esophagogastric junction; RFS, recurrence-free survival; OS, overall survival; 5-YSR, 5-year survival rate;

HR, hazard ratio; CI, confidence interval; TNM, tumor- node- metastasis; TrNM, tumor- node ratio- metastasis.

Moreover, the survival discriminatory ability between the TNM and TrNM staging systems was evaluated by the HR and −2log likelihood. We also found that, compared with the 7th edition TNM staging system, the TrNM staging system was the most appropriate prognostic classification for predicting the RFS (HR: 1.647 *vs*.1.495; −2log likelihood: 3062.115 *vs*. 3078.584) and OS (HR: 1.664 *vs*.1.494; −2log likelihood: 2874.578 *vs*. 2892.807).

### Comparison of the predictive accuracy for the survival of patients with AEG

Finally, the area under the receiver operating characteristic curve (AUC) and the Harrell's concordance index (C-index) values for each staging system were calculated to evaluate the predictive accuracy of the aforementioned staging systems.

**Figure 4:**
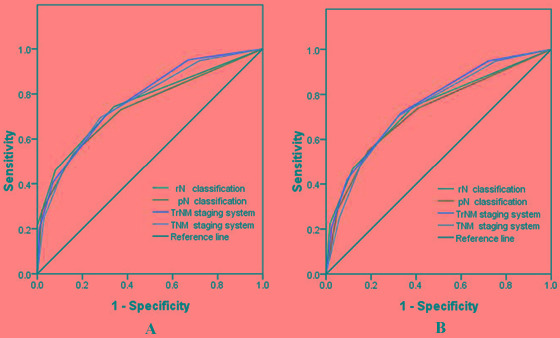
Receiver operating characteristic curves of the pN and rN classifications, TNM and TrNM staging systems for the prediction of the survival of AEG patients after curative surgery **A.**, For RFS; and **B.**, For OS

For RFS, the AUC of the rN and pN classifications were 0.759, 0.738, respectively, the difference was not significantly different (z = 0.582, *P* > 0.05). The AUC for the TrNM and TNM staging system were 0.776, 0.764, respectively, there was also no significantly different (z = 0.293, *P* > 0.05). Furthermore, for OS, the AUC of the rN, pN, TrNM and TNM staging system were 0.736, 0.721, 0.756 and 0.739, respectively, no significantly different was observed among these staging systems (z = 0.400, 0.454, *P* > 0.05, for all) (Table 5, Figure 4).

**Table 5:** Prognostic ability comparison among the different staging systems for patients with AEG

Staging systems	RFS				OS				
	HR	−2log likelihood	AUC	C-index		HR	−2log likelihood	AUC	C-index
rN classification	1.637	3066.658	0.759	0.729		1.646	2879.804	0.736	0.714
pN classification	1.442	3087.341	0.738	0.701		1.448	2899.995	0.721	0.697
TrNM staging system	1.647	3062.115	0.776	0.745		1.664	2874.578	0.756	0.726
TNM staging system	1.495	3078.584	0.764	0.722		1.494	2892.807	0.739	0.701

AEG, adenocarcinoma of the esophagogastric junction; AUC, area under the curve; C-index: concordance index

pN, lymph node status; rN, N-ratio; TNM, tumor- ratio-metastasis; TrNM, tumor- node ratio-metastasis

Similarly, for RFS, the rN and the modified TrNM staging system presented a significantly larger C-index compared with the pN and TNM staging system. Moreover, for OS, the rN and TrNM staging system yielded a significantly larger C-index than the pN and TNM staging system did (Table 5).

These results indicated that the rN and TrNM staging system may have better prognostic stratification and more precise prediction than the pN and TNM staging system for patients with AEG.

## DISCUSSION

An effective tumor staging system is used to predict prognosis and determine the most appropriate multidisciplinary therapeutic modalities. These objectives can be accomplished by applying a feasible, reproducible, and accurate staging system for the prognostic stratification without stage migration of AEG.

The presence of lymph node metastasis has been previously considered as an important prognostic factor, and it has been incorporated into the AJCC TNM staging system [9, 10]. However, in this staging system, the number of metastatic LNs is influenced by the number of removed LNs, which itself depends on several controllable factors, such as surgical procedures or pathological evaluation; and some uncontrollable factors, such as patient and tumor characteristics. In cases few LNs are removed, the N stage cannot be classified accurately. Thus, incorrect pathologic staging would occur and lead to improper treatment. This phenomenon is well-known as stage migration or Will Roger's phenomenon [11].

Although curative resection remains as the cornerstone for the treatment of AEG, the appropriate procedures of surgery, extent of lymphadenectomy and the exact number of LNs should be examined remain unaddressed for this type of tumor; this limitation is attributed to its dependence on tumor location and safety margin length achieving R0 resection [3, 9, 12]. Van Cutsem [13] and Barbour et al [14] suggested that patients with Siewert types II and III AEG should undergo adequate lymphadenectomy with the removal of ≥15 LNs to assess the pN stage precisely. Conducting a large retrospective study, Gee et al [15] stated that preferably 20-25 LNs should be removed to determine the prognosis and treatment for AEG tumors.

The use of rN, which is defined as the ratio between metastatic and examined LNs, has been proposed by several authors. It has been deemed to be a simple, reliable, and reproducible method that can be used to better evaluate the status of lymph node metastasis and to identify the subgroup of patients with esophageal, gastric, breast, and colon cancer with similar prognosis; thus minimizing the “stage migration” phenomenon that can be observed when the conventional TNM staging system is used [16, 17]. Furthermore, it is applicable to cases with insufficient number of retrieved LNs [7, 18, 19] or to cases subjected to limited lymphadenectomy [20, 21].

We found that rN was correlated with the number of metastatic nodes but not with the total number of removed LNs. This finding indicated that a specific number of dissected LNs are required to ensure an accurate assessment of lymph node status. Using rN may significantly reduce the potential bias and minimize stage migration phenomenon because it is slightly influenced by the type of lymphadenectomy and the number of dissected LNs.

However, up until now, there is still no consensus on the optimal categorization with regard to the rN has been reported in previous literatures and the most suitable methods to determine cut-off levels also differ among studies [4, 7, 18, 19, 22]. Liu et al. [19] classified 1,325 esophageal cancer patients into four groups based on rN: 0, 0-0.25, 0.25-0.5, and > 0.5, and confirmed that an increasing rN was linearly associated with a poorer long-term survival than pN category when an insufficient number of LNs were examined. According to a retrospective study involving a series of 710 patients with gastric cancer, the ratio was classified as 0, 1%-10%, 10%-25%, and > 25%. They demonstrated that the N-ratio was a reliable classification that may improve the current nodal staging system and help stratify prognosis regardless of the number of examined LNs [18]. In a study [23] using the database of the Surveillance, Epidemiology and End Results (SEER) data of 18,043 gastric cancer patients subjected to gastrectomy, rN ratio was identified as follows: rN1: 0-1/15, rN 2: 1/15-3/10, rN 3: 3/10-7/10, and rN 4: *>* 7/10; a TrNM staging system was also constructed. The misclassification rate is 12% for the proposed TrNM system. Therefore, the modified TNrM system is more effective and rational than the current TNM system in the staging of gastric cancer in the SEER database. The system can also help doctors determine the patients who can benefit from adjuvant treatments.

In the present study, the ratio was classified by the best cut-off approach as follows: 0, 1-30%, 31-60%, and > 60%. Kaplan-Meier survival analysis revealed that, both pN and rN classifications were significantly associated with RFS and OS. Subsequent multistep multivariate analysis indicated that, either pN or rN can be used as an independent variable for RFS and OS. However, rN remained as an independent prognostic factor and pN lost its significance when both pN and rN categories were introduced as covariates to the same model. This phenomenon is consistent with that reported by other investigators. Furthermore, the subgroups of AEG patients can be identified in more detail and more precision by using the rN category than the N category.

In addition, on the basis of the superiority of rN classification to pN classification, we therefore combined the pT and rN classification to form the hypothetical TrNM staging system and then compared it with the conventional TNM staging system. The survival rate could be easily distinguished between patients when the N classifications were replaced with rN classifications in the staging system. Comparing the −2log likelihood and HR, we found that the proposed TrNM staging exhibited greater discriminatory abilities to predict survival than the traditional TNM staging did. In the predictive accuracy analysis, the most commonly used method is the AUC by ROC analysis [24]. In our study, the AUCs were larger in the rN and TrNM staging system than in the pN and TNM staging system in terms of RFS and OS. Moreover, the rN and TrNM staging system yielded a significantly larger C-index than the pN and TNM staging system did. These findings demonstrated that rN should be recommended as an important variable to enhance the accuracy of the prognostic prediction of AEG patients.

The limitation of the current study is its retrospective analysis setting, including a relatively small sample size from a single center was included in the study. Therefore, a multiple-center clinical trial with a large sample size should prospectively provide further evidence to evaluate the optimal cut-off point of N-ratio. Moreover, the pathological diagnosis of lymph node metastasis was simply analyzed by hematoxylin and eosin staining. No special techniques, such as immunohistochemistry or reverse-transcription polymerase chain reaction, were routinely used to identify micrometastases [25]. In addition, almost half of the patients unlikely gain the benefits of the rN classification for predicting outcomes because the definition of the rN0 classification is consistent with the pN0 classification. Log odds of positive LNs, which is defined as the log of the ratio between the number of positive and negative LNs, has been proposed recently [26, 27]. However, the strength of this study is that we did not limit our analysis to the OS but also included the RFS, which is a specific endpoint in the field of cancer-related investigations. The adoption of this new TrNM staging system may help oncologists predict prognosis, make sound treatment decisions, and facilitate informed discussion with patients in terms of recurrence and death, especially for those who underwent limited lymph node dissection.

In conclusion, we demonstrated that the rN classification is a potentially useful and reliable factor to predict RFS and OS for patients with AEG. Incorporation of rN into the current staging system could help address the limitations of N classification and enable clinicians to predict the prognosis of AEG patients accurately. Nevertheless, further studies should be conducted to overcome the limitations of our study and validate our results.

## PATIENTS AND METHODS

### Study design and patient population

From January 2000 to January 2007, a retrospective database of 389 patients with histologically confirmed adenocarcinoma of the gastroesophageal junction (AEG II-III), who had underwent curative resection and systematic lymphadenectomy in the thoracic surgery and gastrointestinal surgery departments were retrospectively reviewed. This study protocol was approved by the Research Ethics Committee of Tianjin Medical University Cancer Institute and all patients were provided written informed consent for the use of their information in the hospital database.

The eligibility criteria included histologically confirmed R0 resection, which was defined as no macroscopic and microscopic residual tumor and a postoperative survival time of > 3 months. None of these cases received any kind of neoadjuvant treatments, such as preoperative chemotherapy and/or radiotherapy. Type I lesions of the gastroesophageal junction were designated as cancers of the esophagus; and patients with distant metastasis or peritoneal dissemination confirmed during the surgery were excluded from the study. Based on these criteria, 389 patients with types II and III tumors were included in the study. Their demographic and clinical characteristics are shown in Table 1.

Before surgery was performed, endoscopy and barium swallows were performed to determine Siewert type. Chest radiography, cervical and abdominal ultrasonography, and computed tomography (CT) scans from the neck to the upper abdomen were conducted to exclude liver and lung metastasis. Bone scanning was selectively carried out to exclude bone metastasis for patients with symptoms of bone pain. The operation was selected based on preoperative diagnosis and estimated length of esophageal invasion. All of the patients underwent a proximal or total gastrectomy with lymphadenectomy *via* a transthoracic or transabdominal approach.

The resected specimens were histologically examined by the same group of gastrointestinal pathologists in our institution. Histopathological types were classified as differentiated (well/moderately differentiated or papillary adenocarcinoma) or undifferentiated (poorly differentiated or undifferentiated adenocarcinoma, signet-ring cell carcinoma or mucinous adenocarcinoma or other types of tumors) according to the World Health Organization (WHO) classifications [28]. According to Lauren's classification, the cancers were classified as intestinal type and diffuse-mixed type [29]. All patients were staged on the basis of the 7th edition of the American Joint Committee on Cancer (AJCC) TNM staging system for esophagus and esophagogastric junction cancer [10, 30].

### Lymph node classification

Lymph node metastasis was classified according to the 7th edition AJCC N staging system: pN0 refers to no metastasis; pN1 refers to 1~2 positive LNs; pN2 refers to 3~6 positive LNs; and pN3 refers to 7 or more positive LNs.

The N-ratio was calculated as the ratio of the number of metastatic LNs to the total number of removed LNs. rN intervals was determined by the best cut-off approach in terms of the log-rank test. To make the study compatible with the 7th edition of AJCC TNM staging system, we proposed a novel TrNM hypothetical staging system on the basis of the pT classification and the aforementioned rN classifications. The TrNM staging system is as follows: I (IA, T1rN0; IB, T1rN1, T2rN0); II (IIA, T1rN2, T2rN1, T3rN0; IIB, T1rN3, T2rN2, T3rN1, T4arN0); IIIA(T2rN3, T3rN2, T4arN1); IIIB(T3rN3, T4arN2, T4brN0, T4brN1); IIIC(T4arN3, T4brN2, T4brN3). Then, RFS and OS rates based on pN and rN as well as the TNM, TrNM staging were compared.

### Follow-up

After curative resection, all patients were generally monitored every 3 months for the first 2 years, every 6 months during the third to fifth years, and then every year until death or the last follow-up. Check-up items included physical examination, tumor-marker examination, chest radiography, CT scans, neck and abdominal ultrasonography, endoscopic examination, and bone scan when necessary to detect recurrence and/or metastasis. Recurrence of the disease was determined by clinical examination or imaging method. The follow up was completed in September 2012 with a rate of 92.3% and the median follow-up period was 36 months (range, 3~144 months). The cases lost to follow-up were treated as censored data for the analysis of survival rates.

The primary endpoint of the study was recurrence-free survival (RFS), which was defined as the period from surgery to disease progression or recurrence or the date of death or last follow-up. The secondary endpoint was overall survival (OS), which was calculated as the time from operation to the point of death or last follow-up.

### Statistical analysis

Statistical analyses were performed using SPSS 17.0 (SPSS, Inc., Chicago, IL) software and programming languages R (version 3.2.2 for Windows). Spearman correlation analysis was calculated to assess the correlation among the number of retrieved LNs, metastatic LNs and rN. The rN cut-off was determined by the best cut-off approach using log-rank test as previous study reported [18, 27]. The 5-year RFS, OS analyses were calculated on the basis of the Kaplan-Meier method and compared by the log-rank test. Factors deemed as potentially important by univariate analyses ( *P* < 0.05) were subjected to multivariate Cox proportional hazards model to identify the independent prognostic factors. Multivariate survival analyses were performed using the Cox regression proportional hazards model to identify the independent factors. The HR and −2log likelihood values within a Cox regression were calculated for each category to measure its discriminatory ability. The larger the HR, the better the system. While the smaller the −2log likelihood, the better the system. Furthermore, the predictive accuracy of different categories was evaluated using the area under the receiver operating characteristic curve (AUC) and the Harrell's concordance index (C-index). The larger the AUC or C-index, the more precise was the survival prediction [31]. A two-tailed *P* value of < 0.05 was considered to be statistically significant.

## References

[1] Amini N, Spolverato G, Kim Y, Squires MH, Poultsides GA, Fields R, Schmidt C, Weber SM, Votanopoulos K, Maithel SK, Pawlik TM (2015). Clinicopathological features and prognosis of gastric cardia adenocarcinoma: a multi-institutional US study. J Surg Oncol.

[2] Hosoda K, Yamashita K, Katada N, Watanabe M (2015). Overview of multimodal therapy for adenocarcinoma of the esophagogastric junction. Gen Thorac Cardiovasc Surg.

[3] Sisic L, Blank S, Weichert W, Jager D, Springfeld C, Hochreiter M, Buchler M, Ott K (2013). Prognostic impact of lymph node involvement and the extent of lymphadenectomy (LAD) in adenocarcinoma of the esophagogastric junction (AEG). Langenbecks Arch Surg.

[4] Tan Z, Ma G, Yang H, Zhang L, Rong T, Lin P (2014). Can lymph node ratio replace pn categories in the tumor-node-metastasis classification system for esophageal cancer?. J Thorac Oncol.

[5] Barbour AP, Rizk NP, Gonen M, Tang L, Bains MS, Rusch VW, Coit DG, Brennan MF (2007). Lymphadenectomy for adenocarcinoma of the gastroesophageal junction (GEJ): impact of adequate staging on outcome. Ann Surg Oncol.

[6] Wu XJ, Miao RL, Li ZY, Bu ZD, Zhang LH, Wu AW, Zong XL, Li SX, Shan F, Ji X, Ren H, Ji JF (2015). Prognostic value of metastatic lymph node ratio as an additional tool to the TNM stage system in gastric cancer. Eur J Surg Oncol.

[7] Wei C, Deng WY, Li N, Shen W, Zhang C, Liu JY, Luo SX (2015). Lymph Node Ratio as an Alternative to the Number of Metastatic Lymph Nodes for the Prediction of Esophageal Carcinoma Patient Survival. Dig Dis Sci.

[8] Zhao LY, Li CC, Jia LY, Chen XL, Zhang WH, Chen XZ, Yang K, Liu K, Wang YG, Xue L, Zhang B, Chen ZX, Chen JP, Zhou ZG, Hu JK (2016). Superiority of lymph node ratio-based staging system for prognostic prediction in 2575 patients with gastric cancer: validation analysis in a large single center. Oncotarget.

[9] Peng J, Wang WP, Yuan Y, Hu Y, Wang Y, Chen LQ (2015). Optimal Extent of Lymph Node Dissection for Siewert Type II Esophagogastric Junction Adenocarcinoma. Ann Thorac Surg.

[10] Yoshikawa T (2015). [Esophagogastric junction cancer in the TNM classification]. [Article in Japanese]. Nihon Geka Gakkai Zasshi.

[11] Feinstein AR, Sosin DM, Wells CK (1985). The Will Rogers phenomenon. Stage migration and new diagnostic techniques as a source of misleading statistics for survival in cancer. N Engl J Med.

[12] Zhang H, Wang W, Cheng Y, Song Y, Zhu K, Dang C (2013). Adenocarcinomas of the esophagogastric junction: experiences at a single institution in China. World J Surg Oncol.

[13] Van Cutsem E, Van de Velde C, Roth A, Lordick F, Kohne CH, Cascinu S, Aapro M (2008). Expert opinion on management of gastric and gastro-oesophageal junction adenocarcinoma on behalf of the European Organisation for Research and Treatment of Cancer (EORTC)-gastrointestinal cancer group. Eur J Cancer.

[14] Barbour AP, Rizk NP, Gonen M, Tang L, Bains MS, Rusch VW, Coit DG, Brennan MF (2007). Lymphadenectomy for adenocarcinoma of the gastroesophageal junction (GEJ): impact of adequate staging on outcome. Ann Surg Oncol.

[15] Gee DW, Rattner DW (2007). Management of localized esophageal cancer in the older patient. Oncologist.

[16] Chen S, Zhao BW, Li YF, Feng XY, Sun XW, Li W, Zhou ZW, Zhan YQ, Qian CN, Chen YB (2012). The prognostic value of harvested lymph nodes and the metastatic lymph node ratio for gastric cancer patients: results of a study of 1,101 patients. PLoS One.

[17] Chen SB, Weng HR, Wang G, Zou XF, Liu DT, Chen YP, Zhang H (2015). Lymph node ratio-based staging system for esophageal squamous cell carcinoma. World J Gastroenterol.

[18] Lin D, Li Y, Xu H, Chen J, Wang B, Liu C, Lu P, Alatengbaolide (2013). Lymph node ratio is an independent prognostic factor in gastric cancer after curative resection (R0) regardless of the examined number of lymph nodes. Am J Clin Oncol.

[19] Liu YP, Ma L, Wang SJ, Chen YN, Wu GX, Han M, Wang XL (2010). Prognostic value of lymph node metastases and lymph node ratio in esophageal squamous cell carcinoma. Eur J Surg Oncol.

[20] Pedrazzani C, Sivins A, Ancans G, Marrelli D, Corso G, Krumins V, Roviello F, Leja M (2010). Ratio between metastatic and examined lymph nodes (N ratio) may have low clinical utility in gastric cancer patients treated by limited lymphadenectomy: results from a single-center experience of 526 patients. World J Surg.

[21] Maduekwe UN, Lauwers GY, Fernandez-Del-Castillo C, Berger DL, Ferguson CM, Rattner DW, Yoon SS (2010). New metastatic lymph node ratio system reduces stage migration in patients undergoing D1 lymphadenectomy for gastric adenocarcinoma. Ann Surg Oncol.

[22] Lee SR, Kim HO, Son BH, Shin JH, Yoo CH (2012). Prognostic significance of the metastatic lymph node ratio in patients with gastric cancer. World J Surg.

[23] Wang J, Dang P, Raut CP, Pandalai PK, Maduekwe UN, Rattner DW, Lauwers GY, Yoon SS (2012). Comparison of a lymph node ratio-based staging system with the 7th AJCC system for gastric cancer: analysis of 18,043 patients from the SEER database. Ann Surg.

[24] Li XQ, He WP, Hou WH, Chen JW, Fan RR, Yuan LJ, Yang GP, Cai MY, Chen L, Li J, He SY, Xie D, Yang GF, You ZS (2016). Overexpression of RNF2 is positively associated with ovarian carcinoma aggressiveness and indicative of poor patient survival. Oncotarget.

[25] Wlodarczyk J, Mueller J, Wlodarczyk J (2013). Lymph node micrometastases of adenocarcinoma located in gastroesophagal junction. Pol J Pathol.

[26] Wen J, Ye F, He X, Li S, Huang X, Xiao X, Xie X (2016). Development and validation of a prognostic nomogram based on the log odds of positive lymph nodes (LODDS) for breast cancer. Oncotarget.

[27] Wu SG, Sun JY, Yang LC, Zhou J, Li FY, Li Q, Lin HX, Lin Q, He ZY (2015). Prognosis of patients with esophageal squamous cell carcinoma after esophagectomy using the log odds of positive lymph nodes. Oncotarget.

[28] Flejou JF (2011). [WHO Classification of digestive tumors: the fourth edition]. Ann Pathol.

[29] Chen YC, Fang WL, Wang RF, Liu CA, Yang MH, Lo SS, Wu CW, Li AF, Shyr YM, Huang KH (2016). Clinicopathological Variation of Lauren Classification in Gastric Cancer. Pathol Oncol Res.

[30] Suh YS, Han DS, Kong SH, Lee HJ, Kim YT, Kim WH, Lee KU, Yang HK (2012). Should adenocarcinoma of the esophagogastric junction be classified as esophageal cancer? A comparative analysis according to the seventh AJCC TNM classification. Ann Surg.

[31] Fan H, Shao ZY, Xiao YY, Xie ZH, Chen W, Xie H, Qin GY, Zhao NQ (2016). Comparison of the Glasgow Prognostic Score (GPS) and the modified Glasgow Prognostic Score (mGPS) in evaluating the prognosis of patients with operable and inoperable non-small cell lung cancer. J Cancer Res Clin Oncol.

